# The formation and consequences of cholesterol-rich deposits in atherosclerotic lesions

**DOI:** 10.3389/fcvm.2023.1148304

**Published:** 2023-02-28

**Authors:** Frederick R. Maxfield, Noah Steinfeld, Cheng-I J. Ma

**Affiliations:** Department of Biochemistry, Weill Cornell Medicine, New York, NY, United States

**Keywords:** macrophages, lipoproteins, cholesterol, lysosomes, extracellular digestion

## Abstract

Cardiovascular diseases remain the leading cause of death throughout the world. Accumulation of lipoprotein-associated lipids and their interaction with macrophages are early steps in the development of atherosclerotic lesions. For decades, it has been known that aggregates of lipoproteins in the subendothelial space are found in early plaques, and these aggregates are tightly associated with extracellular matrix fibers. Additionally, most of the cholesterol in these subendothelial aggregates is unesterified, in contrast to the core of low-density lipoproteins (LDL), in which cholesteryl esters predominate. This suggests that the hydrolysis of cholesteryl esters occurs extracellularly. At the cellular level, macrophages in early plaques engage with the LDL and ingest large amounts of cholesterol, which is esterified and stored in lipid droplets. When excessive lipid droplets have accumulated, endoplasmic reticulum stress responses are activated, leading to cell death. The cholesterol-laden dead cells must be cleared by other macrophages. For many years, it was unclear how unesterified (free) cholesterol could be formed extracellularly in early lesions. Papers in the past decade have shown that macrophages form tightly sealed extracellular attachments to aggregates of LDL. These sealed regions become acidified, and lysosomal contents are secreted into these compartments. Lysosomal acid lipase hydrolyzes the cholesteryl esters, and the free cholesterol is transported into the macrophages. High concentrations of cholesterol can also lead to formation of crystals of cholesterol hydrate, and these crystals have been observed in atherosclerotic blood vessels. Characterization of this process may lead to novel therapies for the prevention and treatment of atherosclerosis.

## Introduction

With the advent of improved treatment and prevention of infectious diseases and reduction of malnourishment, cardiovascular diseases associated with atherosclerosis are now the leading cause of death worldwide (1–3). Ironically, this growth in mortality has coincided with extraordinary research advances over the past 50 years into the causes and treatment of cardiovascular diseases (4).

Among the advances that have been made are the identification of the low-density lipoprotein (LDL) receptor by Michael Brown and Joseph Goldstein as well as other receptors for lipoproteins (5, 6). Pathways for internalizing lipoproteins, digesting their lipid cores, and transporting cholesterol throughout the cell have also been described (4). Additionally, mechanisms for the regulation of the levels of free cholesterol in cells have been explored in detail (4, 7). These findings led to the widespread acceptance of the importance of controlling circulating cholesterol levels for the prevention of atherosclerosis (4). In addition to control of diet, several pharmacological interventions have been developed that have a significant impact on circulating LDL levels. These include statins, discovered by Akira Endo (8), which block a key early step in the synthesis of cholesterol and have shown significant benefit in long term trials (9). Ezetimibe can block the uptake of cholesterol by intestinal cells by inhibiting the NPC1L1 cholesterol transport protein on these cells (10), and this has been shown to effectively reduce circulating LDL cholesterol (10). A more recent discovery is proprotein convertase subtilisin/kexin type 9 serine protease (PCSK9), which can block the efficient recycling of LDL receptors on liver cells, leading to their degradation (11, 12). This reduction of LDL receptors increases the level of circulating LDL (11). Monoclonal antibodies against PCSK9 can prevent its interaction with LDL receptors, which has been shown to reduce circulating LDL levels (13). RNAi treatments have also been developed that target PCSK9, and one of these, Inclisiran, was approved by the United States Food and Drug Administration in 2021 (14, 15). Small molecule pharmacological agents against PCSK9 are also in development (16).

All of these developments are having a very important effect on reducing disability and death from atherosclerotic diseases. Nevertheless, as noted above, atherosclerotic diseases are still the worldwide leading cause of death. It is likely that more widespread adoption of healthy lifestyles accompanied by more widespread use of therapeutic treatments now available would have a major impact in reducing atherosclerotic diseases. However, it is also true that there are still gaps in our understanding of the causes of atherosclerotic diseases, which opens the opportunity for development of new therapeutic approaches.

## Lipoproteins in the vessel wall

One widely accepted contributor to atherosclerosis is oxidized forms of LDL (Ox-LDL) (17, 18). Macrophages have receptors for Ox-LDL (17), and numerous studies support a role for Ox-LDL in development of atherosclerosis (19, 20). One possibility is that Ox-LDL is endocytosed by scavenger receptors, leading to foam cell formation as a consequence of cholesterol uptake (21). There is some controversy about which receptors are used for endocytosis of Ox-LDL (22, 23). A recent study reported that the transcription factor Nuclear Factor of Activated T-cell isoform c3 (NFATc3) induces expression of microRNA-204 (miR-204), which reduces expression of two receptors reported to bind oxidized lipoproteins, the class A scavenger receptor (SRA) and CD36 (24). Macrophage-specific ablation of NFATc3 increased atherosclerotic plaque formation in a mouse model, while NFATc3 overexpression reduced plaque formation. There was also an inverse correlation between NFATc3 expression in human monocytes and atherosclerosis. An accompanying review suggested that it will be important to follow up by characterizing all the genes whose expression is altered by miR-204 (25).

Studies have indicated that there are effects of minimally oxidized LDL that are dependent on Toll-like receptor 4 (TLR4) and its downstream signaling that result in increased pinocytic lipoprotein uptake and subsequent foam cell formation (18). Interestingly, several of the signaling pathways activated by minimally oxidized LDL, including TLR4, tyrosine kinase SYK, guanine nucleotide exchange factor VAV, phosphatidylinositol 3-kinase (PI3K), and small GTPase CDC42 are also involved in regulation of an alternative lipid uptake process called digestive exophagy (26), which is discussed in detail later. Since several studies testing anti-oxidant treatment found no or only modest benefits (2), it will be important to continue exploring the role of Ox-LDL and how to counter its effects.

Despite years of study, there are some aspects of atherosclerotic plaques that remain poorly characterized. Better understanding of the development and growth of plaques as well as their conversion to disease-associated states may provide new insights for treatment. Mature atherosclerotic lesions are characterized by chronic inflammation, a distorted endothelium, and lipid-laden foam cells that are derived from macrophages and smooth muscle cells. In late stages, there is an accumulation of dead cells, which are remnants of foam cells that were overloaded with stored cholesteryl esters (27).

Low-density lipoproteins, which have a diameter of 22–24 nm, percolate through the endothelium into the interstitial space between smooth muscle cells and the endothelium. The LDL is acted on by lipases, including lipoprotein lipase (28) and sphingomyelinase (29). These changes cause the lipoproteins to aggregate, and they also become tightly linked to the extracellular matrix by bridging proteins including lipoprotein lipase (28, 30–32). It has long been known that the vast majority of LDL in atherosclerotic lesions is tightly bound and not freely soluble (28). It has been suggested that these retained and aggregated lipoproteins are responsible for the inflammation and macrophage foam cell formation in atherosclerosis.

## Interactions between macrophages and lipoprotein aggregates

Macrophages that cross the endothelium rapidly encounter these lipoprotein aggregates (30, 33). It has also long been known that the cholesterol in these retained and aggregated lipoproteins is mostly unesterified, free cholesterol (34–36), which is surprising because nearly all the cholesterol in LDL is esterified. More recently, 3-dimensional electron microscopy studies in human carotid arteries have shown that there are also cholesterol crystals in atherosclerotic lesions, and these are in regions close to macrophages in the interstitial space (33). While it is clear that the cholesteryl esters in the cores of lipoproteins have been hydrolyzed to produce free cholesterol, the mechanism for this hydrolysis has been unclear.

Studies over the past 20 years provide an explanation for this hydrolysis of cholesteryl esters outside the cells and may lead to new insights into the development of atherosclerotic lesions. This new understanding may, in the future, lead to new treatment modalities.

To model the interaction of macrophages with LDL aggregates enmeshed in the extracellular matrix, bovine aortic endothelial or smooth muscle cells were grown to confluency to allow them to form a network of extracellular matrix above the cells. LDL was then added, followed by treatment with sphingomyelinase to induce aggregation and lipoprotein lipase to non-covalently link the aggregated LDL to the extracellular matrix (37, 38). In some experiments the LDL was aggregated by gentle vortexing. The aggregates of LDL formed deep penetrations into the macrophages, and the cholesteryl esters were hydrolyzed much more rapidly than the apoB, which was unexplained at the time. It was demonstrated that the cholesteryl ester hydrolysis was carried out by lysosomal acid lipase, which, at the time, was interpreted to indicate that cholesteryl esters were somehow selectively extracted from the aggregates and delivered to lysosomes (38). These initial studies provided evidence for part of the mechanism by which retained and aggregated LDL contributes to the development of atherosclerosis (28).

**FIGURE 1:**
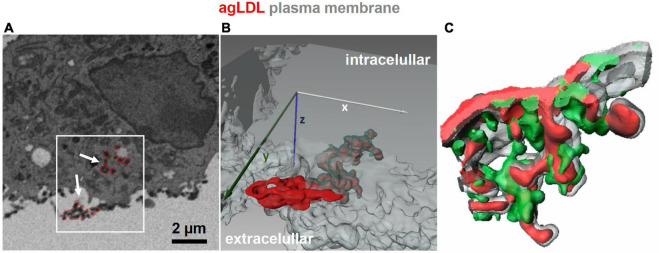
3D imaging of lysosomal synapses. **(A,B)** Focused ion beam-scanning electron microscopy (FIB-SEM) images were acquired from J774 cells incubated with colloidal-gold-labeled aggregated LDL (agLDL) for 1 h. A stack of 100 FIB-SEM images with 40 nm separating each image was obtained, and agLDL was observed within the lysosomal synapse that was connected with the extracellular space. Panel **(A)** shows a single scanning EM image of a macrophage interacting with agLDL. Regions of agLDL labeled with colloidal gold are outlined in red. A 3D representation of the region highlighted by the box in panel **(A)** was generated using image processing software to display agLDL and the cellular plasma membrane forming the compartment. Panel **(B)** shows both extracellular agLDL and the portion contained in deep membrane invaginations. Plasma membrane in the invagination (labeled green) tightly surrounds the agLDL contained within the lysosomal synapse. **(C)** Mouse bone marrow derived macrophages were incubated with Alexa546-agLDL for 1 h, fixed and stained with Alexa488-phalloidin to visualize F-actin. A 3D reconstruction from a confocal microscopy stack shows features such as rings and “fingers” of F-actin (green) surrounding the aggregate (red). The plasma membrane is labeled in gray [adapted from Singh et al. (43)].

More recent studies characterized the interaction of macrophages with aggregated LDL in more detail. It was found that macrophages create tight interactions with the aggregates to allow acidification of the contact region by the vacuolar ATPase, which is found on the plasma membrane of macrophages (39, 40). Secretion of lysosomal contents onto the aggregated LDL was demonstrated by internalizing biotinylated-fluorescein-dextran into lysosomes and attaching streptavidin to the LDL (39). Within 30–90 min, there was abundant release of the lysosomal contents onto the aggregated LDL, as seen by previously lysosomal dextran bound to aggregated LDL outside the cell *via* biotin-streptavidin interactions. There was significant hydrolysis of the cholesteryl esters in the core of the lipoproteins by lysosomal acid lipase, as shown by labeling of the aggregates with filipin, a fluorescent dye that binds to unesterified cholesterol. It was also found that interaction with aggregated LDL triggered polymerization of filamentous actin (F-actin) at or near the contact site. Furthermore, inhibition of cholesteryl ester hydrolysis blocked this actin polymerization (41), indicating that the formation of F-actin was increased by increased cholesterol in the plasma membrane (42). It had been shown previously that F-actin was required for the hydrolysis of cholesteryl esters by macrophages (38). As shown in Figure 1, confocal microscopy and 3-D electron microscopy demonstrated that the aggregated LDL was pulled into deep invaginations in the macrophages, and these were closely associated with F-actin (43). These deep invaginations have been described as “lysosomal synapses,” and the process of extracellular digestion has been called “digestive exophagy” (26). Reactive oxygen species are released from macrophages into the lysosomal synapses (44).

Similar deep invaginations of aggregated LDL into macrophages were observed in an aortic atherosclerotic plaque in an *ApoE^–/–^* mouse on a high fat diet (45). The aggregated LDL stained brightly with filipin, indicating that cholesteryl esters in the core of the aggregated LDL had already been hydrolyzed to free cholesterol while outside the macrophage. The plaque itself was filled with filipin-labeled deposits, confirming previous studies on the composition of lipids in retained LDL in plaques (34–36). These studies in cell culture and *in vivo* show that extracellular hydrolysis of cholesteryl esters in the core of retained and aggregated LDL generates abundant free cholesterol in atherosclerotic plaques. These very high concentrations of cholesterol would lead to formation of cholesterol crystals, which are observed in atherosclerotic lesions (33, 46). These cholesterol crystals can further contribute to inflammatory activation of macrophages (47).

**FIGURE 2:**
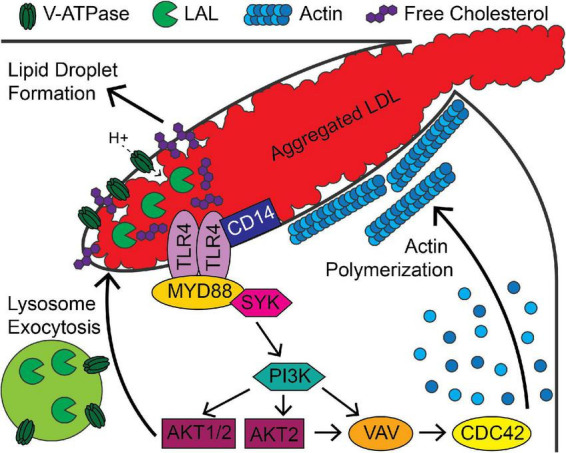
Signaling pathways associated with digestive exophagy of aggregated LDL. Aggregated LDL-induced TLR4/CD14 signaling activates MYD88, PI3K and AKT and leads to formation of the lysosomal synapse. Activation of signaling through AKT1 and AKT2 stimulates lysosome exocytosis, which delivers lysosomal acid lipase (LAL) to the compartment. Vacuolar ATPase (V-ATPase), present on the plasma membrane and the lysosomal membranes, lowers the pH of the compartment. This activates LAL in the compartment, which stimulates agLDL degradation and generation of free cholesterol. This cholesterol inserts into the plasma membrane and further stimulates signaling. The cholesterol is re-esterified in the cell, leading to formation of lipid droplets. The activity of PI3K and AKT2 lead to VAV and CDC42 activation that support longer term stabilization of the compartment by promoting actin polymerization.

The signaling mechanisms that regulate digestive exophagy are partially understood (Figure 2). There have been several studies of the receptors that interact with aggregated LDL. The LDL receptor-related protein (LRP1) has been shown to be involved in the interaction and degradation of aggregated LDL by macrophages (38, 44, 48). Loss of LDL receptor, CD36, or the SCARA1 scavenger receptor did not affect interactions of macrophages with aggregated LDL (38, 44). A partial signal transduction pathway for digestive exophagy of aggregated LDL has been described (26, 44, 49, 50). TLR4 and MYD88 are plasma membrane proteins that appear to initiate a signaling system that includes SYK, PI3K, serine/threonine kinases AKT1&2, VAV, and CDC42. The RHOA/RHO kinases are activated by ceramide, which is generated by acid sphingomyelinase in the lysosomal synapse (50). The increase in ceramide inhibits the actin polymerization around the lysosomal synapse.

Interestingly, much of this signaling pathway has also been described for the enhanced pinocytic activity in macrophages exposed to minimally oxidized LDL (18). Since reactive oxygen species are generated when macrophages contact aggregates formed from non-oxidized LDL (44), it seems quite possible that the responses of aggregated LDL and minimally oxidized LDL are very similar. *In vivo*, both may occur essentially simultaneously.

There are important aspects of the signaling pathways for digestive exophagy that remain unresolved. For example, organelle secretion would certainly involve RAB and SNARE proteins, but knockdowns of several of these proteins in macrophages that are required for lysosome secretion in other situations (51, 52) have no significant effect on lysosome secretion during digestive exophagy. There are certainly several other signaling pathways involved in regulation of digestive exophagy that remain to be explored. It is not entirely clear how free cholesterol is transferred from the aggregated LDL to the macrophage. It appears to enter *via* the plasma membrane, but it is not known if there are any proteins involved in the transfer from the aggregated LDL to the plasma membrane. In a cell culture model, both HDL and β-cyclodextrins can remove cholesterol from aggregated LDL in contact with macrophages (45). This reduces the transfer of cholesterol into the macrophages, leading to a reduction in lipid droplet formation. It has been noted that smooth muscle cells are often in close proximity with macrophages in atherosclerotic lesions (53, 54). It seems plausible that the free cholesterol in the aggregated LDL might also be transferred to the smooth muscle cells leading to lipid droplet formation, but this has not yet been explored.

## Possible new therapeutic approaches

It seems likely that there are valuable therapeutic opportunities based on this enhanced understanding of the mechanisms of catabolism of LDL in atherosclerotic lesions. A fundamental question is whether slowing digestive exophagy in lesions would be beneficial in slowing atherosclerotic progression. This could be achieved, for example, by inhibiting the processes contributing to digestion of cholesteryl esters and transfer of cholesterol to the macrophages. Unfortunately, the signaling pathways identified so far are used widely throughout the body, so they are not good candidates for therapeutics. It is possible that more specific molecules, such as the RABs and SNAREs involved in lysosomal secretion, might be useful therapeutic targets. Another mechanism for reducing digestive exophagy would be to reduce the aggregation of lipoproteins in atherosclerotic lesions as discussed in a recent review (33). Another possibility is to inhibit the retention of aggregated LDL on the extracellular matrix, which is mediated by lipoprotein lipase (28). Inhibition of this binding to the matrix would allow rapid endocytosis or phagocytosis of the LDL-derived lipoproteins. It also seems likely that removal of the free cholesterol from the extracellular aggregates before it can enter the macrophages would be beneficial. As noted above, both HDL and β-cyclodextrins extract cholesterol directly from aggregated LDL. In addition to the possible benefits of HDL in removing cholesterol from lesions, it has been shown that injection of hydroxypropyl-β-cyclodextrin can promote regression of atherosclerotic lesions in mice (55). This promotion of cholesterol efflux by cyclodextrin was independent of ABCA1- and ABCG1-mediated reverse cholesterol transport. A problem with the potential use of cyclodextrins therapeutically is that, as small polar molecules, they are rapidly cleared after injection. However, larger cholesterol-binding molecules such as β-cyclodextrin polymers might be useful for this purpose, and they are being explored as drug carriers (56). One additional possibility would be to use the unique chemical properties of the retained and aggregated lipoproteins to concentrate beneficial drugs, such as anti-inflammatory agents.

## Summary

Recent studies described in this review describe a novel mechanism for macrophage digestion of lipoproteins in arterial vessel walls. Further investigation of this may lead to new therapies for atherosclerosis.

## Author contributions

FM wrote the first draft which was edited and revised by C-IM and NS. All authors contributed to the article and approved the submitted version.
